# Dispersal Ecology of the Beet Armyworm in the Florida Panhandle: Implications for Outbreaks and Insecticide Resistance Spread

**DOI:** 10.3390/insects16111131

**Published:** 2025-11-05

**Authors:** Eduardo Soares Calixto, João Gabriel Tardin de Moraes, Ethan Carter, Isaac L. Esquivel, Silvana V. Paula-Moraes

**Affiliations:** 1 West Florida Research and Education Center, Department of Entomology and Nematology, University of Florida, Jay, FL 32565, USA; joao.g.moraes@ufv.br; 2 IFAS Extension Jackson County Office, University of Florida, Marianna, FL 32448, USA; ethancarter@ufl.edu; 3 Department of Entomology & Nematology, North Florida Research and Education Center, Institute of Food and Agricultural Services, University of Florida, Quincy, FL 32351, USA; isaac.esquivel@ufl.edu; 4 Department of Entomology, University of Nebraska-Lincoln, Lincoln, NE 68583, USA

**Keywords:** beating tray, hydrogen isotopes, insect pests, insecticide resistance, integrated pest management, insect resistance management, pheromone trap, reverse migration, *Spodoptera exigua*

## Abstract

Beet armyworm (BAW), *Spodoptera exigua* (Hübner), is a polyphagous and migratory lepidopteran pest that causes damage to a broad range of host plants, including row crops and vegetables, with increasing reports of insecticide-resistant populations. Understanding its movement ecology is essential for integrated pest management (IPM) and insect resistance management (IRM) programs. We investigated the population dynamics and migratory origins of BAW during the 2024 cropping season in two counties of the Florida Panhandle, USA. Larval sampling and adult pheromone trapping were conducted in commercial peanut fields, and hydrogen stable isotopes were used to infer the probable geographic origins of moths. Across the season, the presence of BAW accounted for 7.1% of all individuals collected, with infestation occurring only in July and August. Moth captures were significantly higher in the eastern Florida panhandle than in the western Florida Panhandle. Stable isotope analysis revealed four distinct migratory patterns, with most BAW likely originating from South Florida, the Caribbean, or the central and southern United States, indicating northward movement. Our findings provide critical insights into the movement ecology of BAW and highlight its implications for outbreak forecasting, resistance management, and regional IPM/IRM strategies in the southeastern United States.

## Simple Summary

The beet armyworm is an insect pest that feeds on many different crops, including peanuts and vegetables, and it has become more difficult to control because some populations are no longer affected by common insecticides. Knowing where these insects come from and how they move is important to reduce crop losses and improve control strategies. In this study, we tracked beet armyworm populations in peanut fields in two counties in the Florida Panhandle during the 2024 growing season. We collected caterpillars and adult moths using traps and also used a chemical marker found in rainwater to help identify where the moths originated. We found that beet armyworm was present only during July and August, making up about seven percent of all insects collected. The eastern county had more moths than the western county. Results showed that most beet armyworms likely came from South Florida, the Caribbean, or the southern United States, moving northward during the season. These findings improve our understanding of beet armyworm movements and will help farmers and scientists better forecast outbreaks and develop more effective and sustainable ways to manage this pest.

## 1. Introduction

Beet armyworm (BAW), *Spodoptera exigua* (Hübner) (Lepidoptera: Noctuidae), is a polyphagous and migratory agricultural pest with a worldwide distribution [1,2,3]. This pest damages leaves, buds, flowers and fruits of vegetables and crops such as peanut, cotton, soybean, tomato, and corn [4,5]. Historically, before the widespread adoption of transgenic Bt cotton in the United States, BAW was recognized as a major cotton pest, causing significant economic losses [6,7]. Today, it remains a key pest of non-Bt crops such as peanut, soybean, and tomato [4,5]. Its high reproductive potential, multivoltine life cycle, and ability to persist in tropical and subtropical regions contribute to local outbreaks and regional dispersal [8].

Chemical control remains the main strategy for BAW management in non-Bt crops [9]. However, frequent insecticide applications, including sprays targeting other pests in other host crops, promote BAW exposure and have led to widespread resistance [9,10,11,12]. This species has evolved resistance to multiple classes of insecticides, including carbamate, avermectin, pyrethroid, organophosphate, benzoylphenylurea, endosulfan, spinosyn, and diamide [9,10,12,13,14,15,16,17]. For instance, diamides are highly effective against lepidopteran pests, but resistance in BAW has been detected in the Florida Panhandle [9,12,17]. Although BAW has historically been a secondary pest in peanut, recent outbreaks in the southern USA, including Georgia and Florida, have caused severe defoliation and raised concerns about insecticide efficacy.

These outbreaks might be closely tied to the movement ecology of BAW, which plays a critical role in linking local pest population surges to the regional spread of insecticide resistance. This species frequently migrates over long distances, with combined abundance monitoring and meteorological analyses indicating that individuals can travel approximately 3500 km from Central Asia to Denmark within about two weeks [18,19]. Migration drives rapid gene flow and can homogenize geographically distant populations, facilitating the spread of insecticide resistance alleles once they arise in local hotspots. In the southeastern USA, the Florida Panhandle and Gulf Coast serve as overwintering refuges, with northward spring migration likely contributing to seasonal infestations in peanut, soybean, and other crops [20]. Understanding the occurrence and movement ecology of BAW in the Florida Panhandle is therefore essential for both outbreak forecast and insecticide resistance management (IRM) on a regional scale. In addition, it can also be used for predicting BAW risk to northern latitudes during the crop season, improving the timing of pest monitoring and insecticide adoption.

In this study, we documented the flight phenology and probability of origin of BAW during the crop season in the Florida Panhandle, USA. Larval scouting was performed in commercial peanut fields, and moths were trapped using a commercial formulation of sex pheromone. The study was conducted on the west and east sides of the Florida Panhandle, in the Santa Rosa (west side) and Jackson (east side) counties, approximately 200 miles apart. Hydrogen stable isotopes were used as biogeochemical markers to infer the probability of origin of the collected individuals [21,22,23]. Overall, our results contribute to a better understanding of the movement ecology of BAW in the Florida Panhandle, an important overwintering region [6,20] and a key hub for the movement of multiple crop pests to northern latitudes in the southeastern USA [22,23,24,25].

## 2. Materials and Methods

### 2.1. Larval and Moth Sampling

Larvae were sampled with a beating tray, from 7 June to 21 August 2024, in four peanut fields in Santa Rosa County (latitude and longitude of field 1: 30.771553, −87.141925; field 2: 30.7798856, −87.1427537; field 3: 30.8242651, −87.0568868; field 4: 30.9519779, −87.0627766). These fields were selected to provide representative coverage of peanut production within the county, and the sampling period encompasses much of the peanut crop season in the region, representing a good cover of the county. In each field, five sampling points were established, each at least 20 m from field edges and other sampling points. Each field was treated as an independent replicate. Larvae were collected on 10 sampling dates in field 1 and 6 sampling dates in fields 2, 3, and 4. Larvae were collected and identified to species.

To monitor seasonal peaks in moth abundance, green bucket traps containing pheromone lures (TRÉCÉ Pherocon beet armyworm (BAW) lures, GL/TR-3210-25, Great Lakes IPM, Vestaburg, MI, USA) were installed 1.5 m above ground, at least 100 m apart, across Santa Rosa (west side) and Jackson (east side) counties in the Florida Panhandle. Traps were checked one or two times per week, and lures were replaced every 4 weeks. In Santa Rosa County, four traps were placed at the West Florida Research and Education Center (30.772987, −87.139373), and in Jackson County, 1–2 traps were installed at the four fields (30.943936, −85.427858; 30.883368, −85.505939; 30.798775, −85.069406; 30.950063, −85.144000). Traps in Santa Rosa County were installed on 20 June 2024 and removed on 4 September 2024, whereas traps in Jackson County were installed on 10 July 2024 and removed on 21 August 2024. Host crops in both locations were mainly represented by soybean, peanut, and cotton. Because trap inspections were conducted at varying intervals (one or two times per week), moth captures were standardized as the number of insects per trap per day for each sampling date. All moth specimens were stored at −20 °C until further analysis. To evaluate if the number of BAW moths captured in the sex pheromone trapping per day was different between the west and east sides of the Florida Panhandle, we used a Generalized Linear Mixed-Effects Model (GLMM) with the number of moths per trap per day as the response variable, county as the fixed effect, and trap ID as the random effect. The model was fit using the *glmmTMB* package [26], Wald χ^2^ test results were obtained using the *car* package [27], and estimated marginal means were computed with the *emmeans* package [28].

### 2.2. Stable Hydrogen Isotopes Analysis

We selected a subsample of 26 moths for hydrogen isotope analysis, 13 from each side of the Florida Panhandle (Table 1), distributed across the data collection. The right forewing of each moth was removed, processed, and analyzed for hydrogen isotopic ratios. The procedure involved brushing wing scales with a fine paintbrush and cleaning the wings with 70% ethanol to remove surface oils, following the methods of Paula-Moraes et al. [22] and Calixto and Paula-Moraes [23]. This approach is consistent with the traditional 2:1 chloroform–methanol wash [29] and has been validated by Calixto and Paula-Moraes [23]. Once dried, the wings were cut into small pieces and analyzed for hydrogen isotopes at the Stable Isotope Mass Spec Lab, University of Florida, Gainesville, FL, USA. Samples and standards were measured using a Thermo Electron DeltaV Plus isotope ratio mass spectrometer (Thermo Fisher Scientific, Waltham, MA, USA) connected to a ConFlo IV interface and a TCEA (high-temperature conversion elemental analyzer). After being weighed and placed into 4 mm x 6 mm silver capsules, samples were stored in 96-well plates for 48 h to ensure isotopic consistency with the standards [30]. Two keratin standards (CBS and KHS) were used to determine nonexchangeable hydrogen.

After loading the capsules into a Zero Blank autosampler at 1400 °C, hydrogen isotopic values (δ^2^H) were measured using a Picarro L2120-I isotopic liquid water and water vapor analyzer (Picarro, Santa Clara, CA, USA), which was connected to a Picarro A0211 high-precision vaporizer and a CTC HTS PAL autosampler (Picarro, Santa Clara, CA, USA). Measurement precision was determined using USGS42, yielding a value of 2.99‰ (N = 11). To standardize the results, two internal water standards from the University of Florida (UW Antarctic water and Lake Tulane water) were used, both calibrated against international standards (USGS49 and USGS50). Isotopic values are reported in standard delta notation relative to Vienna Standard Mean Ocean Water. Further details on the methodology are available in Paula-Moraes et al. [22] and Calixto and Paula-Moraes [23].

### 2.3. Inferences on the Probability of Origin

The probability of origin for each moth was determined following the methodology outlined in Ma et al. [31] using the package *assignR* in the R software version 4.3.1 [32]. First, we used hydrogen isotope values from amount-weighted, growing-season precipitation at a 5 arc-minute resolution to build an isoscape encompassing a geographic area from 10° N to 50° N latitude and 130° W to 50° W longitude, covering North America as well as parts of the Caribbean and Central America. Then, this isoscape was calibrated using known hydrogen isotope values obtained from published literature to establish the relationship between environmental hydrogen isotopes (e.g., from rainfall) and those in wing tissue (calibration equation: y = −56.14 + 0.61x, R^2^ = 0.73). Finally, the probability of each sample originating from different locations within the isoscape was assessed, and the average distance and direction of potential origins were calculated. Dispersal direction and distance were estimated using the wDist function, which calculates weighted distances and bearings between each sample’s collection site and potential origin locations based on posterior probability surfaces. To define likely origin regions, we applied a probability threshold, extracting the 10% (90% confidence interval) of grid cells with the highest posterior probability for each sample using the qtlRaster function. Details of inferences on the probability of origin of moths can be found in Calixto and Paula-Moraes [23].

## 3. Results

### 3.1. Larvae Recovered

**Figure 1:**
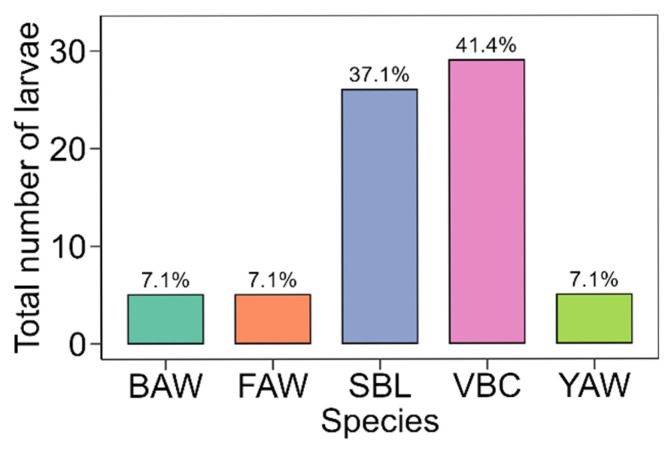
Total number of larvae collected in commercial peanut fields in the FL Panhandle, USA, using the beat cloth method. BAW—*Spodoptera exigua*, FAW—*S. frugiperda*, SBL—*Chrysodeixis includens*, VBC—*Anticarsia gemmatalis*, YAW—*S. ornithogalli*.

**Figure 2:**
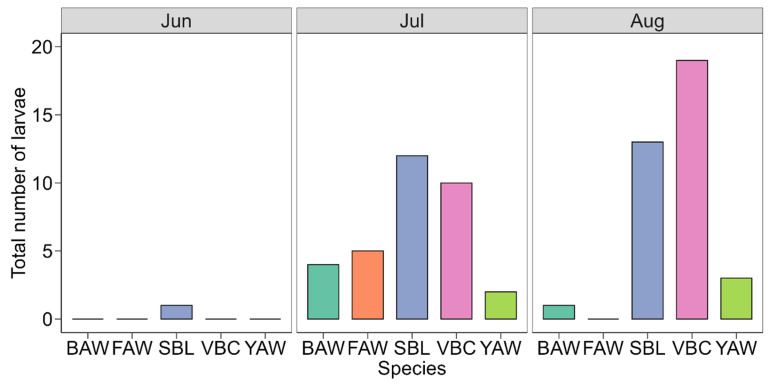
Total number of larvae collected in commercial peanut fields in the FL Panhandle, USA, over the crop season using the beat cloth method. BAW—*Spodoptera exigua*, FAW—*S. frugiperda*, SBL—*Chrysodeixis includens*, VBC—*Anticarsia gemmatalis*, YAW—*S. ornithogalli*.

A total of 70 larvae were recovered from the peanut sampling (Figure 1). Larvae of BAW were detected only in July and August, with four larvae collected in July (12.1%) and one in August (2.8%) (Figure 2). Other larvae, such as *Spodoptera frugiperda* (fall armyworm, FAW), occurred only in July (15.2%). *Anticarsia gemmatalis* (velvet bean caterpillar, VBC) and *Chrysodeixis includens* (soybean looper, SBL) were the predominant species, accounting for 41.4% and 37.1% of all larvae, respectively (Figure 1). Larval abundance was highest in July, driven primarily by SBL (36.4%) and VBC (30.3%), and peaked again in August with VBC representing 52.8% of all larvae (Figure 2). Only one *S. ornithogalli* (yellow-striped armyworm, YAW) was collected in June, and the species remained at low abundance throughout the season. Overall, the larval density per field was low, with 0.4 ± 0.7 (mean ± standard deviation) BAW per field, 0.4 ± 1 FAW per field, 2.2 ± 2.3 SBL per field, 2.4 ± 5.4 VBC per field, and 0.4 ± 0.7 YAW per field.

### 3.2. Pheromone Trapping

**Figure 3:**
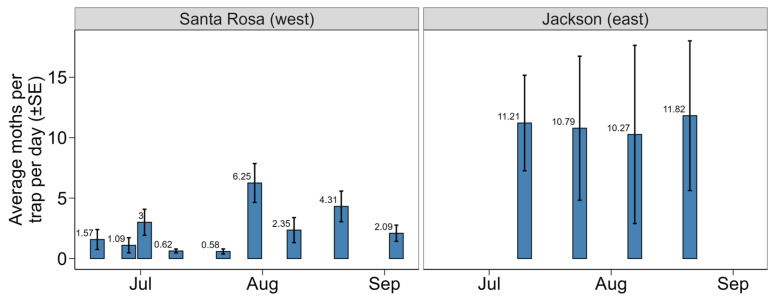
Average number of BAW moths per trap per day collected in pheromone traps on the west (Santa Rosa County) and east side (Jackson County) of the Florida Panhandle, USA. Bars represent the mean with standard error. Numbers above each bar represent the average number of moths.

Significant difference in the number of BAW moths captured in the sex pheromone trapping per day was detected between the west and east sides of the Florida Panhandle (χ^2^ = 21.92, df = 1, *p* < 0.001; Figure 3). Average captures were 4.53 times higher in Jackson County (east) (11.02 ± 1.53 SE) compared to Santa Rosa County (west) (2.43 ± 1.02 SE). Temporal trends showed consistently high captures in Jackson County throughout July and August, whereas Santa Rosa County exhibited lower and more variable captures, peaking on 30 July 2024 with 6.2 ± 1.6 SE moths per trap per day (Figure 3).

### 3.3. Probability of Origin

**Figure 4:**
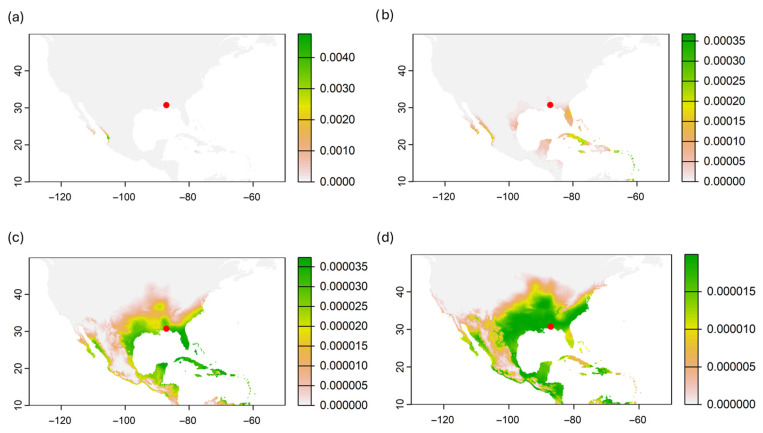
Probability of origin of BAW collected in the Florida Panhandle region, FL. USA, based on hydrogen isotope ratios (δ^2^H). Green represents higher probability of origin. Each map represents an example of the dispersal pattern of a single BAW moth specimen. Red dot represents where the moth was collected. Maps of the probability of origin of all samples are in Figures S1–S3.

**Figure 5:**
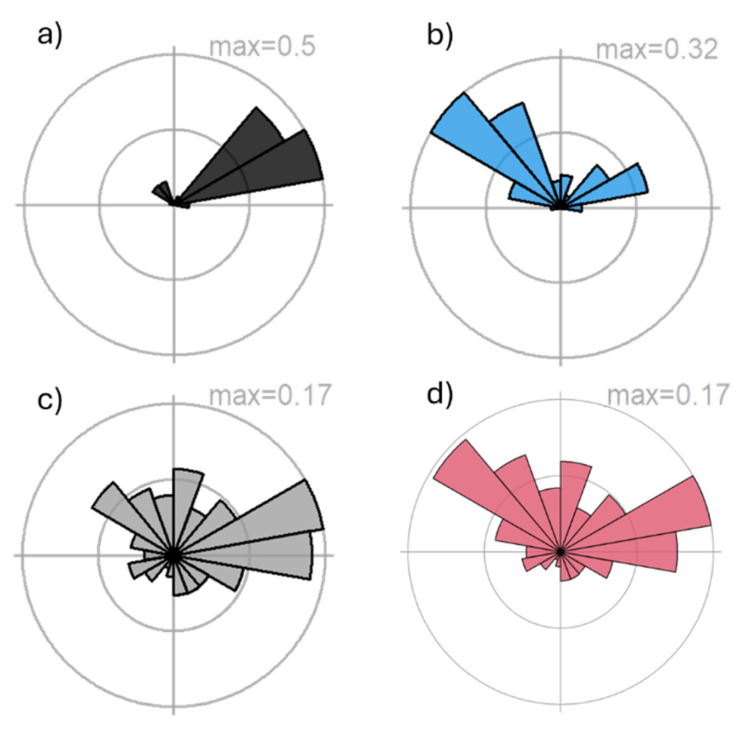
Four patterns of the likely dispersal direction of BAW moths collected in the Florida Panhandle region, FL, USA. Rose diagrams of the likely dispersal direction and distance of all samples are in Figures S4 and S5.

We identified four distinct patterns in the probability of origin for BAW when using hydrogen isotopes (Figure 4 and Figure S1–S3). The first pattern, observed in five samples (19.2% out of 26 samples), suggests a potential origin in the Gulf of California, northwestern Mexico, or the Caribbean region (Figure 4a). Rose diagram bars indicate movement from the southwest (Figure 5a and Figure S4), with an average distance of 2056 ± 52 km (mean ± SD) and an average bearing of 39 ± 15° (Table S1, Figure S5). The second pattern, represented by seven samples (26.9%), indicates a higher probability of origin in South Florida, the Caribbean, and the Gulf of California (Figure 4b and Figure S1–S3) compared to Pattern 1. The average bearing for the probability of origin is 350 ± 3°, suggesting potential movement from the south and southeast (Table S1, Figure S5), with an average distance of 1651 ± 100 km (Figure 5b and Figure S4).

The third pattern, which includes most of the samples (12, 46.1%, Figures S1–S3), shows a high probability of origin in the southern USA and the Caribbean region, particularly in South Florida and the Caribbean (Figure 4c). The average distance from the probable origin of the moths is 1243 ± 16 km (Figure 5c and Figure S4), with an average bearing of 27 ± 7° (Table S1, Figure S5), suggesting movement primarily from the west and southwest. However, some potential movement from the southeast is also indicated by the rose diagram (Figure 4c and Figure S4). Finally, one sample represents a fourth pattern, suggesting a high probability of origin in the central and southern USA, with lower probabilities in South Florida and most of the Caribbean region (Figure 4d and Figure S3). The distance from the probable origin is 1263 km (Figure 5d and Figure S4), with a bearing of 125° (Table S1, Figure S5). Although this direction suggests movement from the southeast, the high probability of origin in central and southern USA states also indicates the possibility of a southward movement (Figure 4d and Figure 5d).

## 4. Discussion

Understanding the movement ecology of agricultural pests is crucial for predicting and forecasting outbreaks, promoting timely pest detection, management tactic adoption, and ultimately enhancing crop yields. Our study provides novel insights into the seasonal occurrence and potential migratory patterns of BAW in the Florida Panhandle. This region is in the northwestern portion of the state and is recognized as a key overwintering area and source of migratory moths for the southeastern USA [6,20,33].

Across the crop season, we collected a total of 70 larvae in peanut fields, representing five pest species, with BAW accounting for only a minor component of the larval community. Larvae of BAW were detected exclusively in July and August, coinciding with the mid-season of peanut and soybean production in the region. Although recent outbreaks have been reported in peanut crops in Florida and Georgia, our sampling on the west side of the Florida Panhandle resulted in very low larval abundance. This pattern is consistent with a previous study in the western Florida panhandle that documented the first case of BAW resistance to diamides [9] and may reflect the delayed population buildup in this area following annual dispersal from southern overwintering regions.

Our pheromone trap data confirms that moths were present in Santa Rosa County (western Florida panhandle) as early as June. However, the average trap capture per day was substantially lower than that of Jackson Co. (eastern Florida panhandle), which recorded 4.5 times more captures. June temperatures were within the optimal developmental range for BAW [34], with mean daily values of 25.6 °C (21.6–31 °C, min-max respectively), while the reported optimal range for larval development and survival lies between 27–35 °C [34]. This suggests that thermal conditions were suitable for local development, but the relatively reduced adult activity in Santa Rosa County likely constrained local population establishment, contributing to the low larval counts observed in commercial fields. Additionally, BAW populations in the northern Gulf region can overwinter locally during mild winters, despite the absence of a true diapause [6]. However, in areas where persistent overwintering does not occur, local populations typically remain low and depend on annual migration from southern regions to initiate infestations. Historical observations from Louisiana and other southeastern states support this pattern, where isolated fields experienced sporadic infestations from June to August that often coincided with regional outbreaks in neighboring states [35,36]. These records suggest that local overwintering populations, when present, may serve as foci for early-season activity, but widespread outbreaks in the northern Gulf typically rely on migration and favorable seasonal conditions to build damaging populations.

Stable hydrogen isotope analysis revealed four distinct patterns of probable origin, reflecting a mixture of local and long-distance migratory individuals. Although the number of moths used to infer the probability of origin was low, most samples (Patterns 2 and 3, ~73% combined, Figure 4) originated from South Florida and the Caribbean, strongly supporting the hypothesis that the Florida Panhandle acts as a sink for northward migration from overwintering populations in these southern regions [22,23,24,25]. Long-distance movements from northwestern Mexico or the Gulf of California were inferred for a subset of individuals; however, this scenario is unlikely due to multiple geographic barriers, the regional landscape, and prevailing wind patterns. These moths also showed low probability values in the Caribbean, which we consider a more plausible source region for this migratory pattern. Finally, one sample suggested a potential southward (reverse) migration, consistent with the bidirectional seasonal movement observed in other noctuid species such as fall armyworm (*S. frugiperda*) [23,37,38] and corn earworm (*Helicoverpa zea*) [22,39,40,41]. These findings highlight that the Florida Panhandle supports a dynamic mix of resident and migratory BAW populations.

The seasonal influx of migratory BAW to the Florida Panhandle has direct implications for both outbreak prediction and insecticide resistance management. First, the timing of larval detections and adult abundance coincides with the mid-season development of peanut and soybean, when crop canopies provide suitable oviposition sites and host quality supports rapid population growth. This seasonal alignment may contribute to the sporadic but damaging outbreaks historically observed in the southeastern USA. Second, the detection of probable origins highlights the risk of resistance allele movement across regions. Previous studies have documented *S. frugiperda* resistance to chemical insecticides in the southeastern USA [42,43], and BAW resistance in the Florida Panhandle [9,12]. In the southern USA, in addition to peanut and soybean, vegetables such as brassica crops and tomato also serve as hosts for BAW, sustaining populations year-round and acting as sources for infestations in the Florida Panhandle [5]. In addition to chemical insecticides, resistance to the Vip3Aa Bt toxin in cotton has also been reported in North Carolina, increasing selection pressure by Bt traits [44]. Therefore, if moths are moving from high-input regions with high pressure of insecticides, migratory movement could facilitate the spread of these alleles between localities, complicating regional resistance management strategies.

The combination of low local larval abundance, high adult captures, and hydrogen isotope evidence of long-distance dispersal suggests that the Florida Panhandle functions as a migratory hub for BAW. Variation in moth captures between the two counties under study was observed. These counties represent the west and east sides of the Florida Panhandle, and the spatial heterogeneity in adult activity is likely driven by differences in local population buildup and the influence of distinct migratory pathways. These findings provide a foundation for future IPM programs that should couple movement ecology with resistance management, ultimately improving the sustainability of crop protection in the southeastern USA.

## Figures and Tables

**Table 1:** Hydrogen isotope ratio of 26 moths collected during the crop season in the west and east sides of the Florida Panhandle, USA, Santa Rosa and Jackson counties, respectively.

Sample ID	δ^2^H	Date Collection	Florida County
1	−28.22	20 June 2024	Santa Rosa
2	−21.85	20 June 2024	Santa Rosa
3	−18.19	20 June 2024	Santa Rosa
4	−44.59	20 June 2024	Santa Rosa
5	−46.32	22 July 2024	Santa Rosa
6	−59.11	22 July 2024	Santa Rosa
7	−61.52	22 July 2024	Santa Rosa
8	−63.84	22 July 2024	Santa Rosa
9	−63.93	21 August 2024	Santa Rosa
10	−59.62	21 August 2024	Santa Rosa
11	−63.56	21 August 2024	Santa Rosa
12	−33.45	21 August 2024	Santa Rosa
13	−44.12	21 August 2024	Santa Rosa
14	−40.56	10 July 2024	Jackson
15	−33.38	10 July 2024	Jackson
16	−39.04	10 July 2024	Jackson
17	−44.41	10 July 2024	Jackson
18	−71.02	7 August 2024	Jackson
19	−47.52	7 August 2024	Jackson
20	−65.97	7 August 2024	Jackson
21	−32.70	7 August 2024	Jackson
22	−65.41	7 August 2024	Jackson
23	−62.40	21 August 2024	Jackson
24	−59.18	21 August 2024	Jackson
25	−60.17	21 August 2024	Jackson
26	−59.84	21 August 2024	Jackson

## Data Availability

The original contributions presented in this study are included in the article/Supplementary Materials. Further inquiries can be directed to the corresponding author.

## Supplementary Materials

The following supporting information can be downloaded at https://www.mdpi.com/article/10.3390/insects16111131/s1. Figure S1: Probability of origin of adults of BAW (samples 1 to 9) collected in the Florida panhandle, FL, USA, based on hydrogen isotope ratios (δ2H). Green color represents higher probability of origin; Figure S2: Probability of origin of adults of BAW (samples 10 to 18) collected in the Florida panhandle, USA, based on hydrogen isotope ratios (δ^2^H). Green color represents higher probability of origin; Figure S3: Probability of origin of adults of BAW (samples 19 to 26) collected in the Florida panhandle, USA, based on hydrogen isotope ratios (δ^2^H). Green color represents higher probability of origin; Figure S4: Likely dispersal direction of BAW moths collected in the Florida panhandle, USA; Figure S5: Likely dispersal distance of BAW moths collected in the Florida panhandle, USA; Table S1: Likely dispersal distance and direction based on the posterior probability of origin for each BAW sample collected in the Florida Panhandle, Jay, FL, USA. Data represent the average and percentiles (10th, 50th, and 90th).

## Abbreviations

The following abbreviations are used in this manuscript:

BAW	Beet armyworm
Bt	*Bacillus thuringiensis*
FAW	Fall armyworm
IPM	Integrated Pest Management
IRM	Insect Resistance Management
SBL	Soybean looper
VBC	Velvet bean caterpillar
YAW	Yellow-striped armyworm
