# Supplementary material for: Dispersal Ecology of the Beet Armyworm in the Florida Panhandle: Implications for Outbreaks and Insecticide Resistance Spread
Source: Insects. 2025 Nov 5;16(11):1131. doi: 10.3390/insects16111131 (PMC12653066; doi:10.3390/insects16111131)
Supplement: Supplementary file 1 [file insects-16-01131-s001.zip › insects-3921530-supplementary.pdf]

Dispersal ecology of the beet armyworm in the Florida Panhandle: implications for outbreaks and insecticide resistance spread

Eduardo Soares Calixto [1], João Gabriel T Moraes [1], Ethan Carter [2], Isaac L. Esquivel [3], Silvana V. Paula-Moraes[4*]

[1] West Florida Research and Education Center, Department of Entomology and Nematology, University of Florida, Jay, FL 32565, USA; calixtos.edu@gmail.com

[2] IFAS Extension Jackson County Office/University of Florida, Marianna, FL, USA; ethancarter@ufl.edu

[3] Entomology and Nematology Department, North Florida Research and Education Center, IFAS/University of Florida, Quincy, FL, USA; isaac.esquivel@ufl.edu

[4] Department of Entomology, University of Nebraska-Lincoln, Lincoln, NE 68583; silpaulamoraes@gmail.com

*Corresponding author: SVPM - silpaulamoraes@gmail.com

Supplementary

Table S1 – Likely dispersal distance and direction based on the posterior probability of origin for each BAW sample collected in the Florida Panhandle, Jay, FL, USA. Data represent the average and percentiles (10th, 50th, and 90th).

Sample_ID	Hydrogen value	Mean Dist	10 Dist	50 Dist	90 Dist	Mean Bear	10 Bear	50 Bear	90 Bear
1	-28.22	2102.2	1646.4	2068.3	2436.1	44.4	-38.0	59.4	67.0
2	-21.85	2114.8	2031.9	2069.0	2395.0	56.6	56.1	59.1	65.8
3	-18.19	2110.5	2034.2	2067.1	2386.3	58.3	56.8	58.8	65.1
4	-44.59	1619.1	645.7	1470.4	2543.6	-6.5	-57.4	-35.1	68.5
5	-46.32	1563.6	618.0	1400.3	2519.3	-7.7	-58.4	-36.0	69.5
6	-59.11	1265.6	430.3	1178.7	2189.4	18.3	-72.2	11.2	109.9
7	-61.52	1242.3	413.8	1164.5	2154.0	26.2	-76.6	29.4	124.2
8	-63.84	1228.0	403.1	1153.1	2131.9	33.4	-83.4	54.6	139.8
9	-63.93	1231.1	406.9	1155.8	2134.0	32.4	-84.3	52.8	137.8
10	-59.62	1258.7	430.4	1169.0	2180.8	20.1	-72.3	13.0	111.9
11	-63.56	1232.1	406.3	1154.0	2136.2	32.4	-81.8	52.7	138.0
12	-33.45	1995.0	1233.7	2061.1	2499.7	24.3	-47.6	57.6	67.1
13	-44.12	1623.4	644.1	1485.6	2526.2	-6.5	-57.1	-35.6	68.4
14	-40.56	1759.4	758.2	1654.1	2574.0	-1.7	-54.0	-33.2	67.6
15	-33.38	2003.5	1238.6	2057.2	2519.7	23.1	-48.9	57.6	67.3
16	-39.04	1836.5	876.2	1995.4	2901.7	2.2	-53.3	-31.9	67.5
17	-44.41	1620.9	640.4	1470.4	2558.4	-7.9	-57.4	-36.2	68.0
18	-71.02	1263.5	445.8	1208.7	2118.7	125.9	13.7	99.8	-105.3
19	-47.52	1534.5	603.1	1381.8	2506.5	-6.2	-59.8	-35.5	70.3
20	-65.97	1227.8	406.2	1156.9	2121.9	39.4	-91.3	63.1	149.3
21	-32.70	2013.7	1270.5	2063.6	2498.5	26.7	-46.2	58.0	67.3
22	-65.41	1222.6	408.2	1151.3	2117.5	38.0	-87.9	61.2	147.3
23	-62.40	1231.8	401.2	1153.7	2147.7	28.8	-80.3	43.8	130.9
24	-59.18	1271.4	439.4	1183.0	2193.2	18.4	-71.4	11.1	109.5

25	-60.17	1257.9	428.8	1174.6	2173.0	21.7	-74.2	15.1	114.7
26	-59.84	1251.6	420.6	1171.3	2166.2	20.4	-73.7	13.1	114.4

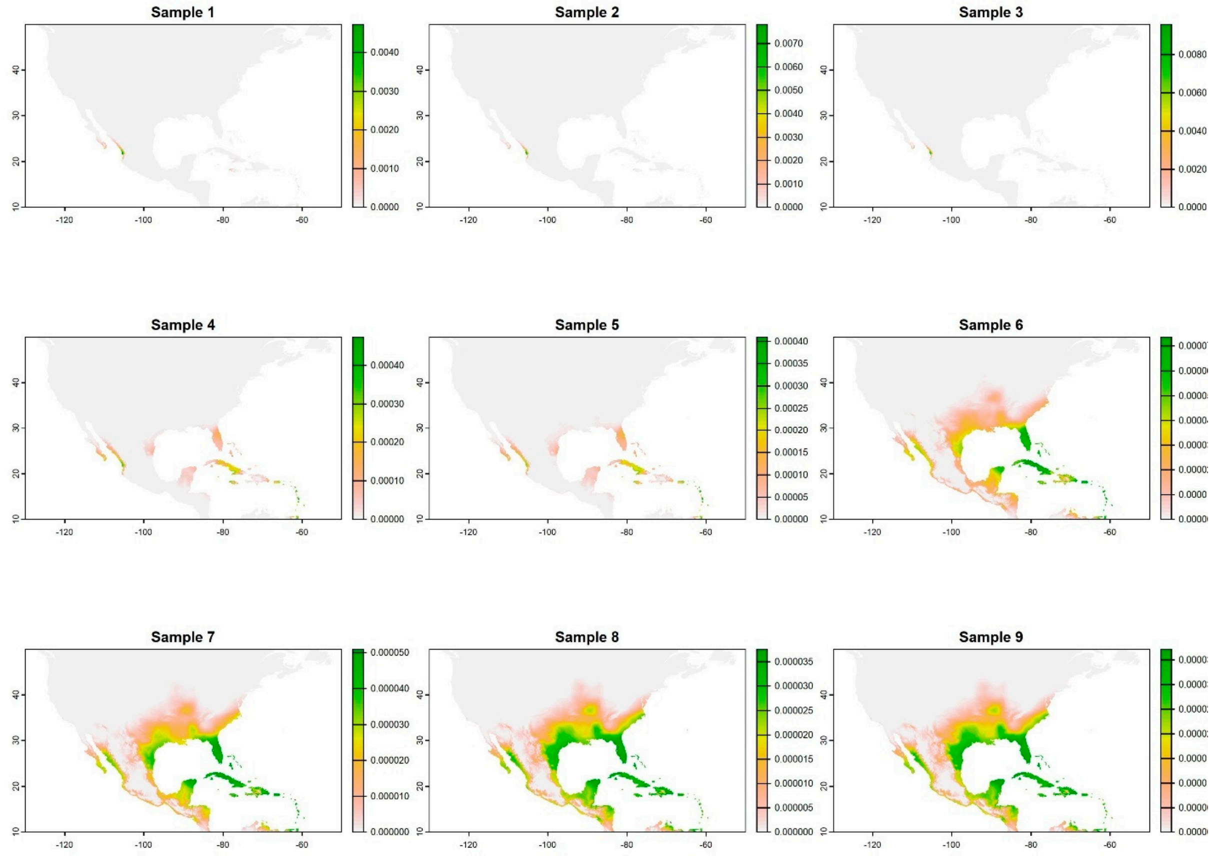

Figure S1 – Probability of origin of adults of BAW (samples 1 to 9) collected in the Florida panhandle, FL. USA, based on hydrogen isotope ratios (δ^2H). Green color represents higher probability of origin.

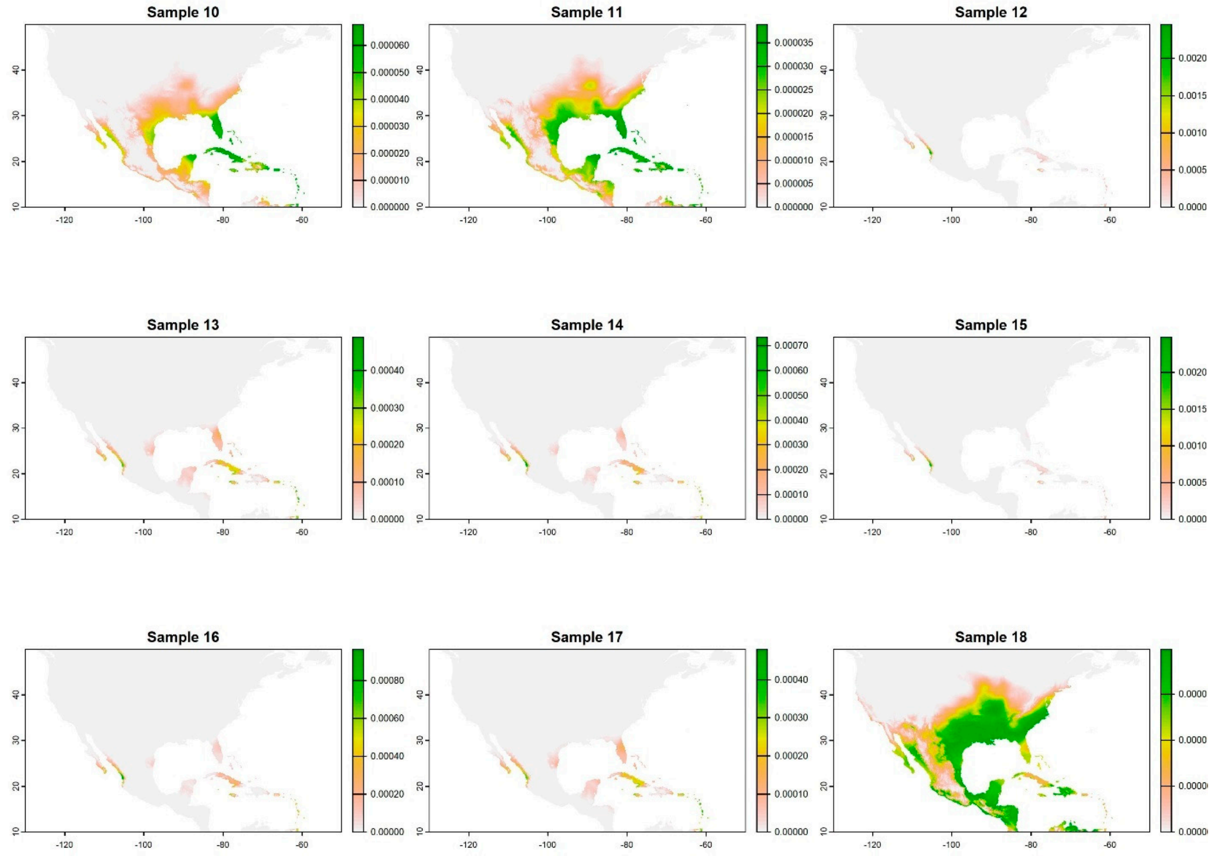

Figure S2 – Probability of origin of adults of BAW (samples 10 to 18) collected in the Florida panhandle, USA, based on hydrogen isotope ratios (δ^2H). Green color represents higher probability of origin.

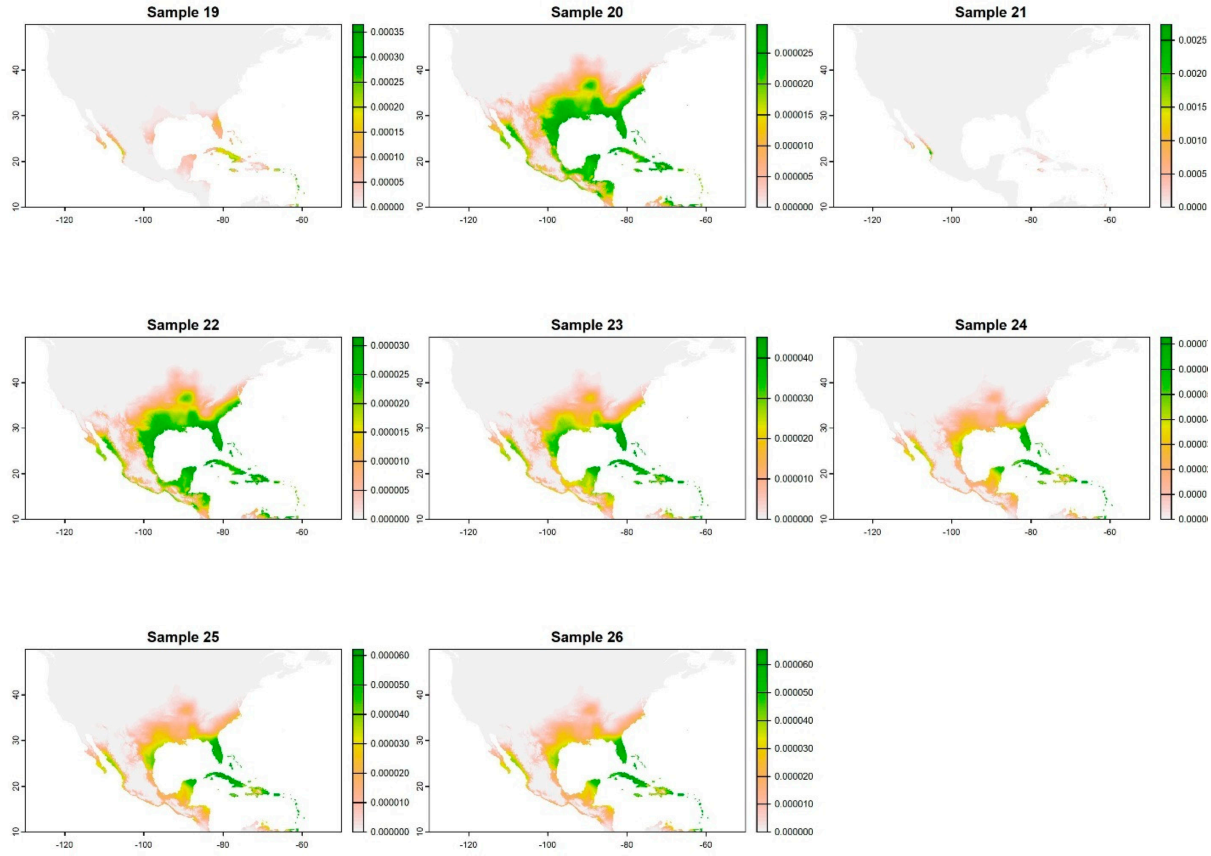

Figure S3 – Probability of origin of adults of BAW (samples 19 to 26) collected in the Florida panhandle, USA, based on hydrogen isotope ratios (δ^2H). Green color represents higher probability of origin.

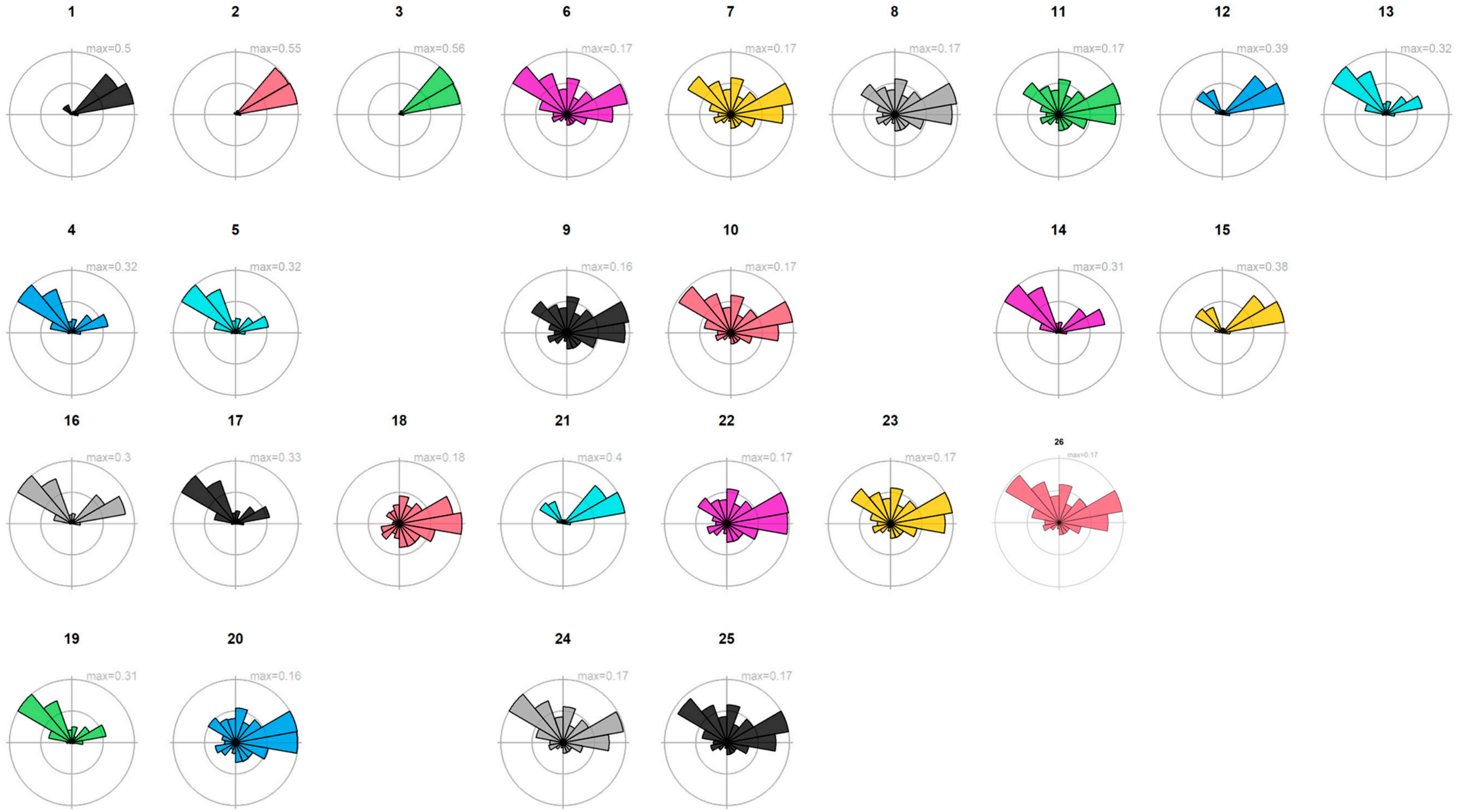

Figure S4 – Likely dispersal direction of BAW moths collected in in the Florida panhandle, USA.

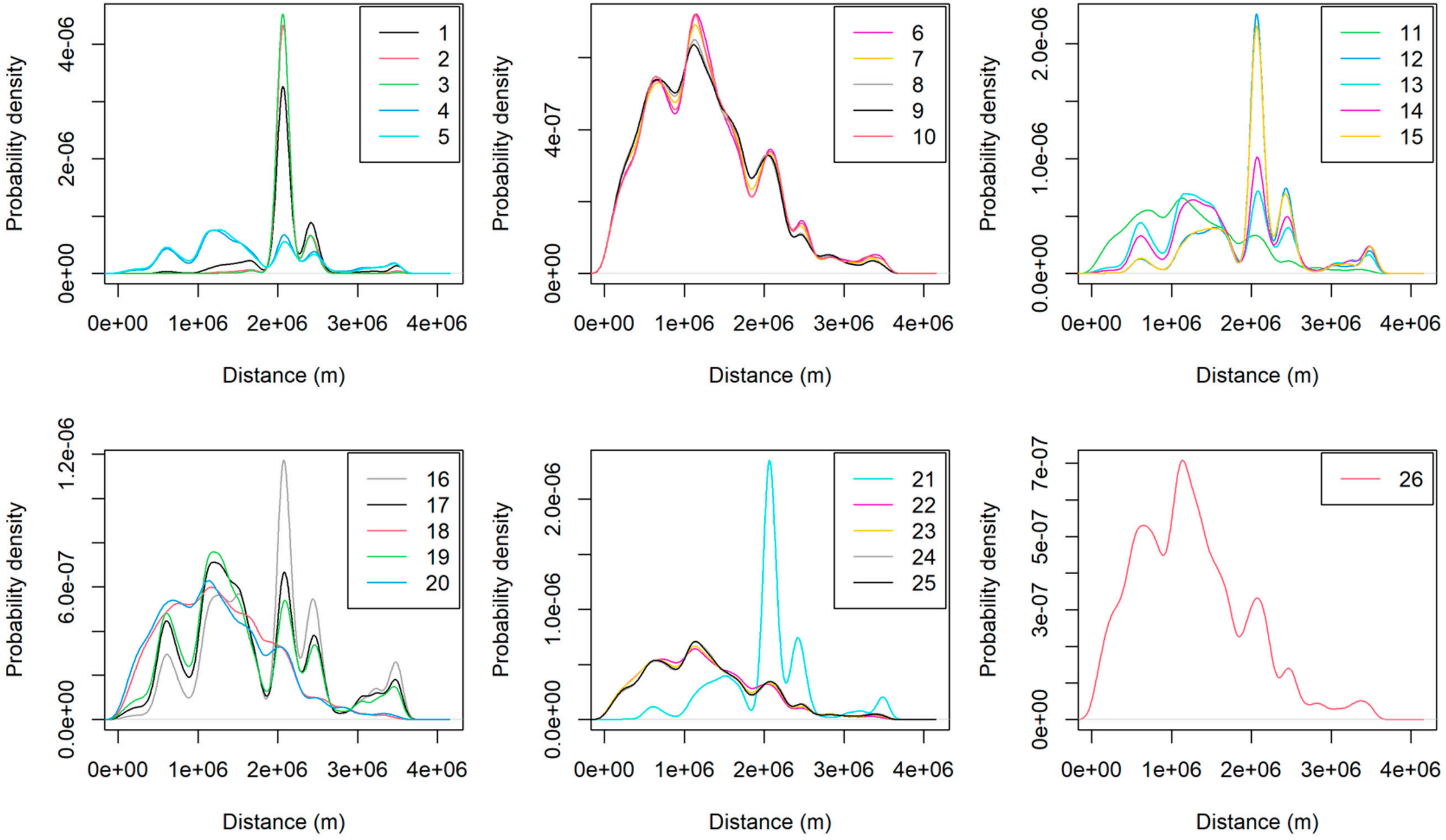

Figure S5 – Likely dispersal distance of BAW moths collected in in the Florida panhandle, USA.
